# Evaluation of Antioxidant Activity, Total Flavonoids, Tannins and Phenolic Compounds in *Psychotria* Leaf Extracts

**DOI:** 10.3390/antiox3040745

**Published:** 2014-11-10

**Authors:** Anelise Samara Nazari Formagio, Carla Roberta Ferreira Volobuff, Matheus Santiago, Claudia Andrea Lima Cardoso, Maria do Carmo Vieira, Zefa Valdevina Pereira

**Affiliations:** 1 Faculty of Agricultural Sciences, University Federal of Grande Dourados (UFGD), 79804-970/533 Dourados-MS, Brazil; E-Mail: mariavieira@ufgd.edu.br; 2 Biological and Environmental Sciences, University Federal of Grande Dourados (UFGD), 79804-970/533 Dourados-MS, Brazil; E-Mails: volobuff81@ gmail.com (C.R.F.V.); matheus_ss@hotmail.com (M.S.); zefapereira@ufgd.edu.br (Z.V.P.); 3 Chemistry Program, State University of Mato Grosso do Sul (UEMS), Mato Grosso do Sul, 79804-970/351 Dourados-MS, Brazil; E-Mail: claudia@uems.br

**Keywords:** *Psychotria*, antioxidant activity, *p*-coumaric acid

## Abstract

The antioxidant activity of *Psychotria carthagenensis*, *P. leiocarpa*, *P. capillacea* and *P. deflexa* (Rubiaceae) extracts were investigated, and the concentrations of total phenolics, flavonoids, condensed tannins and flavonols were determined. The chemical compositions of the extracts were investigated using the high performance liquid chromatography (HPLC/PAD) method. We used 1,1-diphenyl-1-picrylhydrazyl free radical (DPPH), β-Carotene bleaching and 2,2-azinobis (3-ethylbenzothiazoline-6-sulfonic acid) (ABTS) radical cations to determine antioxidant activity. The ability to scavenge radical was measured in these experiments by the discoloration of the solution. Concentrations of constituents were measured spectrophotometrically. *P. carthagenensis* and *P. capillacea* exhibited the highest antioxidant activity, in the DPPH test, β-carotene bleaching and ABTS system. The highest phenolic, flavonoid, condensed tannin and flavonol concentration was found in *P. carthagenensis* and *P. capillacea* extracts. HPLC-PDA analysis of *P. carthagenensis* and *P. capillacea* revealed hydroxycinnamic acid (*p*-coumaric acid). This is the first report on the antioxidant properties and constituent analysis of these *Psychotria* extracts.

## 1. Introduction

Antioxidants, molecules with a radical-scavenging capacity, are thought to exert a protective effect against free radical damage. These biomolecules may contribute to the prevention of many chronic diseases, such as cancer, cardiovascular disease, atherosclerosis, diabetes, asthma, hepatitis and arthritis [1,2]. The consumption of traditional diets prepared with spices and medicinal and aromatic herbs has attracted increasing interest among consumers and scientists because these spices and herbs exhibit antioxidant properties attributed to a variety of bioactive phytochemicals [3,4].

The species of the genus *Psychotria* are used by the population in the form of infusion and with external application. The internal uses are indicated for diseases of the treat disorders gastrointestinal, bronchial diseases and reproductive disorders. In external use, in applications, skin tumors, ulcers, ocular disorders, such as poultices, and baths for the treatment of fever, sore head and ear [5,6]. Reported phytochemical studies on the *Psychotria* genus showed alkaloids, mainly polypirrolidinoindole [7,8,9,10,11,12,13], quinolines [14,15,16,17,18,19], and monoterpene indole alkaloids [20,21,22,23,24,25,26]. Some of these alkaloids display pharmacological effects such as inhibition of human platelet aggregation [27], cytotoxicity [28], and analgesic activity [29], as well as antimalarial and antileishmanial effects [30].

*Psychotria leiocarpa* Cham. and Schlecht., popularly known as “*grandiúva-de-anta*” or “*cafeeiro-do-mato*”, is an understorey woody shrub native to the forests of Southern Brazil [8]. From leaves, was isolated an *N*-glycosylated monoterpenoid indole alkaloid *N*, β-d-glucopyranosyl vincosamide, constitutes up to 2.5% of the dry weight in leaves [31], and iridoid glucosides asperuloside and deacetylasperuloside [32]. Study showed that this alkaloid may act indirectly in *P. leiocarpa* protection against oxidative stress generated upon wounding, UV exposure, and perhaps other environmental stresses [33]. *Psychotria carthagenensis* Jacq., popularly known as “*cafeeiro-do-mato*”, “*carne-de-vaca*” or “*erva-de-rato-branca*”, occurs in most parts of the southern Brazilian State, Rio Grande do Sul. This plant is one of the components of the hallucinogenic beverage *ayahuasca*, used by the people from the Amazonian Forest [34]. Dimetyl-tryptamine was identified as the major component of a leaf extract [35]. *Psychotria deflexa* DC., popularly known as “*erva-de-rato*” or “*café selvagem*”, occurs from Mexico to Argentina. Chemical investigation has previously reported the alkaloid indole chromophore. *Psychotria capillacea* Müll. Arg. Standl., popularly known as “coffee,” occurs in the Brazil states Amazonas, Mato Grosso do Sul, and Parana, as well as in Paraguay and Argentina [24]. The pharmacological properties and chemical for this species was not reported on in the consulted literature.

However, the few biological studies for the species indicate the importance of the continuity of phytochemical studies and activities, with these species reported. Thus, this study represents the first antioxidant investigation of *Psychotria carthagenensis*, *P. leiocarpa*, *P. deflexa* and *P. capillacea* with a different analytical methodology. We also determined the concentrations of phenolic compounds, condensed tannins, flavonoids, and flavonol.

## 2. Experimental Section

### 2.1. General Information

2,4-Dinitrophenylhydrazine (DNPH), 1,1-Diphenyl-2-picrylhydrazyl (DPPH), butylated hydroxyltoluene (BHT), 2,2-azinobis(3-ethylbenzothiazoline-6-sulfonic acid) (ABTS), 6-hydroxy-2,5,7,8-tetramethylchroman-2-carboxylic acid (trolox), quercetin, catechin, β-carotene and *p*-coumaric acid (98%) were purchased from Sigma Chemical Co., (St. Louis, MO, USA). Potassium persulfate, tween 40, Folin-Ciocalteau, and sodium carbonate were purchased from Dinamina (Diadema, Brazil). Sulfuric acid, methanol, ethanol, hydrochloric acid, ascorbic acid, chloroform, linolenic acid, gallic acid, aluminum chloride, sodium acetate, and vanillin were obtained from Vetec (Duque de Caxias, Brazil). Spectroscopic grade acetonitrile was purchased from Merck (Darmstadt, Germany). Standards (caffeic acid (98%), *p*-coumaric acid (98%), luteolin (98%), quercetin (98%) and apigenin (95%)) were purchased from Sigma Chemical Co. (St. Louis, MO, USA).

### 2.2. Plant Material

Leaves were collected in Dourados, Mato Grosso do Sul, Brazil. Botanical identification was performed by Dr. Zefa Valdevina Pereira (Faculty of Biological Sciences and Ambiental, Federal University of Grande Dourados (UFGD)). Specimens of *P. carthagenensis* (DDMS 5006), *P. deflexa* (DDMS 5005), *P. leiocarpa* (DDMS 5007) and *P. capillacea* (DDMS 5008) were deposited in the Herbarium of the Faculty of Biological Sciences and Ambiental, Federal University of Grande Dourados (UFGD), Mato Grosso do Sul, Brazil.

### 2.3. Preparation of Extracts

Dried leaves of *P. carthagenensis* (710 g), *P. deflexa* (620 g), *P. leiocarpa* (560 g) and *P. capillacea* (660 g) were exhaustively extracted by maceration with methanol (8 × 4 L) at room temperature. After filtration, evaporation of the solvent under vacuum furnished the extract. The extract yields (% dry weight) of the *P. carthagenensis*, *P. deflexa*, *P. leiocarpa*, and *P. capillacea* samples were 20.00%, 18.50%, 18.00% and 21.75% (w/w), respectively.

### 2.4. Measurement of Antioxidant Activity

#### 2.4.1. β-Carotene/Linoleic Acid Method

The β-carotene solution was prepared by dissolving 2 mg β-carotene in 10 mL chloroform; 1 mL of this β-carotene-chloroform solution was mixed with 20 mg linoleic acid and 0.2 g Tween 40. Subsequently, the chloroform was removed by a rotary evaporator at 45 °C. Distilled water (50 mL) was slowly added with vigorous agitation to form an emulsion. Emulsion aliquots (5 mL) were transferred with 0.2 mL of the extracts different concentrations (10–200 μg/mL, sample stock 1.0 mg/mL). Control samples were prepared with 0.2 mL methanol devoid of extract [36,37,38]. As soon as the emulsion was added to each tube, absorbance was read at 470 nm against blank (zero time). Tubes were placed in a water bath at 50 °C, and oxidation was monitored by absorbance at 15 min intervals until the color of β-carotene in the control sample had disappeared (105 min). BHT was used as reference. The analyses were performed in triplicate. Antioxidant activity (AA) was calculated as percent inhibition relative to the control:

%AA = [1 − (*Ai* − *At*)/(*A'i* − *A't*)] × 100 (1)

*Ai* = absorbance of sample at zero time, *At* = absorbance of sample after incubation (105 min) at 50 °C, *A'i* = absorbance of control at zero time, and *A't* = absorbance of control after incubation (105 min) at 50 °C.

#### 2.4.2. Scavenging of 1,1-Diphenyl-2-Picrylhydrazyl (DPPH)

Sample stock solutions (1.0 mg/mL) were diluted to final concentrations of 200, 125, 50, 25, 10 and 5 μg/mL in methanol. Samples were added to 3 mL of methanolic DPPH (0.1 mM), prepared daily. The mixture was shaken and left to stand at room temperature in the dark. After 30 min, absorbance was measured at 517 nm against a blank containing all reagents except the test samples [39]. BHT was used as the positive control. Assays were carried out in triplicate. The percentage of inhibition of DPPH (I%) was calculated using the following equation:

I% = (*A*_0_ − *A*/*A*_0_) × 100 (2)

*A*_0_ is the absorbance of the blank solution and *A* is the absorbance of the methanolic extract.

The IC_50_, the concentration giving 50% inhibition of DPPH, was read off a graph of I% (percentage inhibition) *versus* extract concentration.

#### 2.4.3. ABTS^+^ Scavenging Activity

Total antioxidant activity was measured using an improved azinobis (ethylbenzothiazoline-6-sulphonic acid) (ABTS) radical scavenging method [40] with minor modifications. Sample stock solutions (1.0 mg/mL) were diluted to final concentrations of 250, 125, 50, 25, 10 and 5 μg/mL in methanol. Briefly, 7.0 mM ABTS and 140 mM potassium persulphate were mixed and kept in the dark for 16 h at ambient temperature. Before usage, the ABTS^+^ solution was diluted to get an absorbance of 0.700 ± 0.05 at 734 nm with ethanol (P.A.). Then, 3 mL of ABTS^+^ solution was added to 30 μL of different sample concentrations (5–250 μg/mL). After 30 min, the absorbance was taken at 734 nm using spectrophotometer. The ABTS^+^ scavenging activity was calculated using the following equation:

ABTS radical scavenging activity (%) = (*A*_0_ − *A*/*A*_0_) × 100 (3)

*A*_0_ is the absorbance of the blank solution and *A* is the absorbance of the methanolics extracts.

### 2.5. Concentrations of Constituents

#### 2.5.1. Total Phenol Concentration

The total phenol concentration of the samples was determined using folin reagent [41]. Briefly, 100 μL of extract in methanol (1 g/L) were mixed with 1.0 mL of distilled water and 0.5 mL of folin-ciocaleu’s reagent (1:10 v/v). After mixing, 1.5 mL of 2% aqueous sodium bicarbonate were added, and the mixture was allowed to stand for 30 min with intermittent shaking. The absorbance was measured at 765 nm using a spectrophotometer. Total phenolic concentration is expressed as gallic acid equivalent in mg per gram of extract. The methanol solution was used as a blank. All assays were carried out in triplicate.

#### 2.5.2. Total Flavonoid Concentration

The amount of total flavonoids in the extracts was measured spectrophotometrically as previously reported [42]. Briefly, 500 μL of each extract was mixed with 1.50 mL of 95% ethanol, 0.10 mL of 10% aluminium chloride (AlCl_3_.6H_2_O), 0.10 mL of sodium acetate (NaC_2_H_3_O_2_.3H_2_O) (1 M) and 2.80 mL of distilled water. After incubation for 40 min, absorbance was measured at 415 nm using a spectrophotometer. To calculate the concentration of flavonoids, we prepared a calibration curve using quercetin as standard. The flavonoid concentration is expressed as quercetin equivalents in mg per gram of extract. All assays were carried out in triplicate.

#### 2.5.3. Condensed Tannin Concentration (CT)

CT concentrations were determined by a modified version of a method reported previously [41,42]. Samples were mixed with 5 mL vanillin-HCl (8% conc. aq. HCl and 4% vanillin in methanol). Absorbance at 500 nm was read after 20 min. Catechin was used as the standard. The condensed tannin concentration is expressed as catechin equivalents in mg per gram of extract.

#### 2.5.4. Flavonol Concentration

Total flavonols in the plant extracts were estimated using the method reported previously [41,43]. To 2 mL of sample, 2 mL AlCl_3_ (2%)/ethanol and 3 mL (50 g/L) sodium acetate were added. The mixture was shaken and incubated for 2.5 h at 20 °C. Absorbance was read at 440 nm. Total flavonols are expressed as mg of quercetin equivalents per gram of dry weight (mg QE/g extract) using the calibration curve with quercetin.

### 2.6. HPLC/PDA Analysis

The *Psychotria* extracts and standards were analyzed using an analytical HPLC (Varian 210, Varian, Sugar Land, TX, USA) system, with a ternary solvent delivery system and an auto-sampler. A photodiode array detector (PAD) was monitored at λ = 200–800 nm. The HPLC column was C-18 (25 cm × 4.6 mm; particle size, 5 μm; Luna, Phenomenex, Torrance, CA, USA), with a small pre-column (2.5 cm × 3 mm) containing the same packing to protect the analytical column. The flow rate and injected volume were 1.0 mL min^−1^ and 10 μL, respectively. All chromatographic analyses were performed at 22 °C. The elution of *P. leicocarpa* and *P. deflexa* extracts and standards was carried out using acetonitrile with formic acid (pH 2.64) (solvent A) and water (solvent B). The solvent gradient program was as follows: 0 min, 70% B; 30 min, 37% B; in 45 min returning to the initial condition. The *P. carthagenensis* and *P. capillacea* extracts and standards were eluted using acetonitrile with formic acid (pH 2.64) (solvent A) and water (solvent B). The solvent gradient program was as follows: 0 min, 84% B; 15 min, 59% B; 20 min, 79% B; 40 min, 0% B, in 50 min returning to the initial condition.

Stock solutions of standards (caffeic acid, *p*-coumaric acid, luteolin, quercetin and apigenin), created by dissolving individual solutions in acetonitrile at 10 μg/mL and were employed in the identification of compounds using a PDA detector (200–800 nm). This did not reveal coeluting substances.

## 3. Results and Discussion

### 3.1. Antioxidant Activity

Due to the complexity of some plant extracts, the use of several different methods is recommended for the evaluation of antioxidant activity [43]. Currently used methods include the DPPH (1,1-diphenyl-2-picrylhydrazyl) assay [35], which measures the ability of a substance to scavenge the DPPH radical, reducing it to hydrazine. When a substance that acts as a donor of hydrogen atoms is added to a solution of DPPH, hydrazine is obtained, with a change in color from violet to pale yellow. 2,2-azinobis(3-ethylbenzothiazoline-6-sulfonic acid) (ABTS^+^) is a decolorization technique, in that the radical is generated directly in a stable form prior to reaction with putative antioxidants, which involves the production of the blue/green ABTS^+^ chromophore through the reaction of ABTS with potassium persulfate. The β-carotene bleaching method [44] evaluates the ability of a substance to prevent the oxidation of β-carotene, protecting it from the free radicals generated during the peroxidation of linoleic acid.

**Figure 1:**
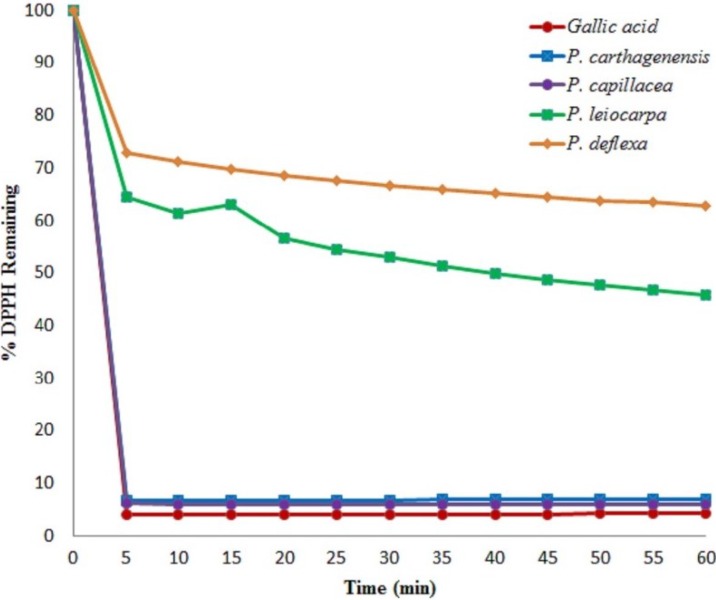
Kinetic behavior of methanol extracts (100 μg/mL) against DPPH: *P. carthagenensis*, *P. leiocarpa*, *P. capillacea*, *P. deflexa* and gallic acid standard.

The effects of methanolic extracts of *Psychotria* species in the DPPH, linoleic acid peroxidation and ABTS assays are shown in Table 1. *P. carthagenensis* and *P. capillacea* exhibited the highest scavenging activity with an IC_50_ of 16.92 ± 4.58 and 30.05 ± 6.22 μg/mL, respectively (Table 1), which was comparable to that of the standard antioxidant butylated hydroxytoluene (BHT) (IC_50_ = 16.72 ± 1.34 μg/mL) and ascorbic acid (IC_50_ = 22.28 ± 0.53 μg/mL). The comparison of the obtained free-radical scavenging data indicated potent activity for the *P. carthagenensis* and *P. capillacea* at 100 μg/mL, with %FRS values of 93.52% ± 8.41% and 91.78% ± 4.23%, respectively. These results show that the DPPH radical scavenging activity of these extracts was similar to BHT and ascorbic acid (Table 1). Gallic acid (6.56%, standard), *P. carthagenensis* (6.47%) and *P. capillacea* (7.32%) also resulted in a remarkable reduction of the DPPH remaining, compared with *P. leicocarpa* and *P. deflexa* (56.26% and 63.51%), respectively, when recorded after 60 min (Figure 1). It is clear that the more DPPH that remains, the lower the radical-scavenging activity of the tested samples is. All these data clearly indicate that *P. carthagenensis* and *P. capillacea* extracts are effective electrons or hydrogen atoms donor to DPPH.

Higher antioxidant activity was also found for *P. carthagenensis* (79.1% ± 3.70%) with the β-carotene bleaching method compared to quercetin and BHT (Table 1). This higher activity was not observed for ascorbic acid, which has relatively high polarity. We propose that this extract contains lipophilic compounds, which act by inhibiting or retarding oxidation of β-carotene.

The scavenging effect of extracts from *Psychotria* and standard on ABTS^+^ decreased in the order: BHT ~ *P. carthagenensis* ˃ *P. capillacea* ~ ascorbic acid ˃ *P. deflexa* ~ *P. leicocarpa* (96.4% ± 2.44%, 92.5% ± 7.43%, 87.34% ± 8.32%, 80.9% ± 5.56%, 15.58% ± 5.22% and 12.20% ± 4.44%, respectively) at the same concentration (100 μg/mL). *P. carthagenensis* and *P. capillacea* exhibited effective radical cation scavenging activity, when compared to other tested extracts.

**Table 1:** Antioxidant activity of *Psychotria* leaf extracts by DPPH, β-carotene/linoleic acid and ABTS assays.

		Test			
		DPPH		β-Carotene/Linoleic Acid	ABTS^+^
		IC_50_ (μg/mL) (Limit Confidence 95%)	%FRS *	(%AA)	(%)
Extracts	*P. carthagenensis*	16.92 ± 4.58	93.52 ± 8.41	79.12 ± 3.70	92.5 ± 7.43
		(12.03–22.71)			
	*P. leiocarpa*	127.00 ± 10.55	54.13 ± 11.10	22.30 ± 7.14	12.20 ± 4.44
		(123.50–135.94)			
	*P. capillacea*	30.05 ± 6.22	91.78 ± 4.23	33.40 ± 15.22	87.34 ± 8.32
		(26.68–37.27)			
	*P. deflexa*	146.40 ± 12.47	66.37 ± 8.12	26.05 ± 10.60	15.58 ± 5.22
		(141.81–148.42)			
Standards	BHT	16.72 ± 1.34	92.19 ± 1.29	91.20 ± 4.54	96.4 ± 2.44
		(14.08–17.22)			
	Ascorbic acid	22.28 ± 0.53	96.40 ± 0.27	4.13 ± 1.42	80.9 ± 5.56
		(20.20–23.43)			
	Quercetin	n.d.	n.d.	80.65 ± 1.25	n.d.

Values are expressed as the mean ± SD (*n* = 3); n.d. = not determined; IC_50_ = concentration resulting in 50% inhibition of DPPH, derived from the graph of I% (inhibition percentage) *versus* concentration in μg/mL; % FRS = free-radical scavenging percentage (* antioxidant activity evaluated by DPPH free-radical scavenging at a final concentration equivalent to 100 μg/mL of extract); %AA = antioxidant activity, evaluated by the β-carotene/linoleic acid method. (%) = ABTS radical scavenging activity.

### 3.2. Levels of Constituents

**Figure 2:**
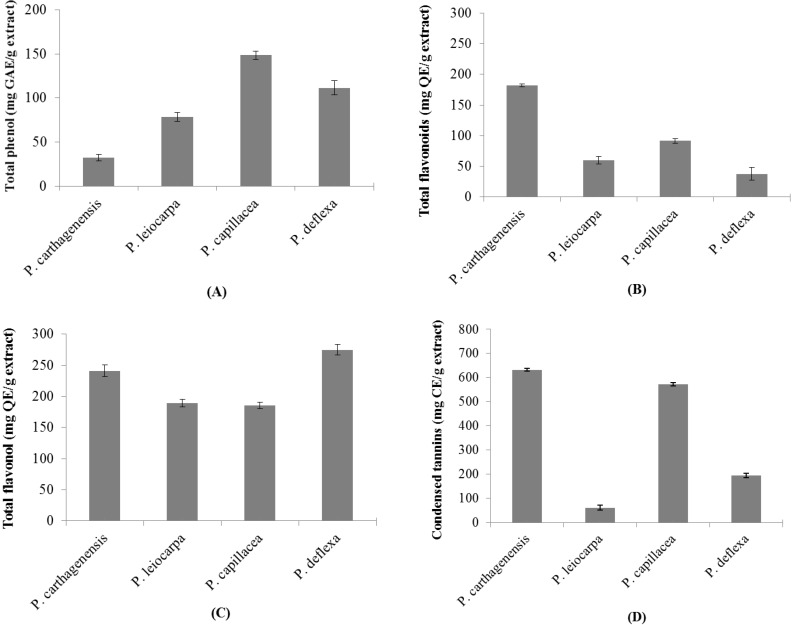
Total phenols (**A**), Total flavonoids (**B**), Total flavonols (**C**) and condensed tannins (**D**) in *P. carthagenis*, *P. leiocarpa*, *P. capillacea* and *P. deflexa* extracts. The data represent the mean ± SD.

The total concentrations of phenolic compounds in the extracts are shown in Figure 2A. *P. capillacea* had the highest total concentration (148.42 ± 4.69 mg gallic acid equivalents (GAE)/g extract), followed by *P. deflexa* and *P. leiocarpa* (111.42 ± 8.12 and 78.45 ± 5.20 mg GAE /g extract, respectively). *P. carthagenensis*, with 182.07 ± 2.78 mg quercetin equivalents (QE)/g extract, exhibited the highest flavonoid concentration (Figure 2B). The flavonoid concentrations of *P. capillacea*, *P. leiocarpa*, and *P. deflexa* were 91.58 ± 3.74, 59.80 ± 6.45, and 37.64 ± 10.14 mg QE/g extract, respectively (Figure 2B). The highest flavonol concentrations were found in extracts *P. deflexa* and *P. carthagenensis* yielded 275.07 ± 8.40 and 241.19 ± 9.48. *P. leiocarpa* and *P. capillacea* also yielded 189.20 ± 6.44, and 185.54 ± 5.33 mg QE/g extract, respectively (Figure 2C). High levels of condensed tannins were also found in *P. carthagenensis* (632.39 ± 5.63 mg catechin equivalents (CE)/g extract) and *P. capillacea* (571.95 ± 7.22 mg CE/g extract), while the lowest concentrations were observed in *P. deflexa* and *P. leiocarpa* (194.67 ± 9.02 and 60.97 ± 10.45 mg CE/g extract, respectively (Figure 2D).

In plants, flavonoids occasionally occur as aglycones, although the most common forms are glycoside derivatives. These compounds account for 60% of total dietary phenolic compounds [45,46]. Flavonols are the most common flavonoids in the plant kingdom, and glycosides of quercetin are the most common naturally occurring flavonols [45].

Phenolic compounds are known as high-level antioxidants because of their ability to scavenge free radicals and active oxygen species, such as singlet oxygen, superoxide free radicals and hydroxyl radicals [47]. The radical-scavenging activity is attributed to replacement of hydroxyl groups in the aromatic ring systems of the phenolic compounds as a result of their hydrogen donating ability [44].

### 3.3. HPLC/PAD Analysis from Psychotria Extracts

**Figure 3:**
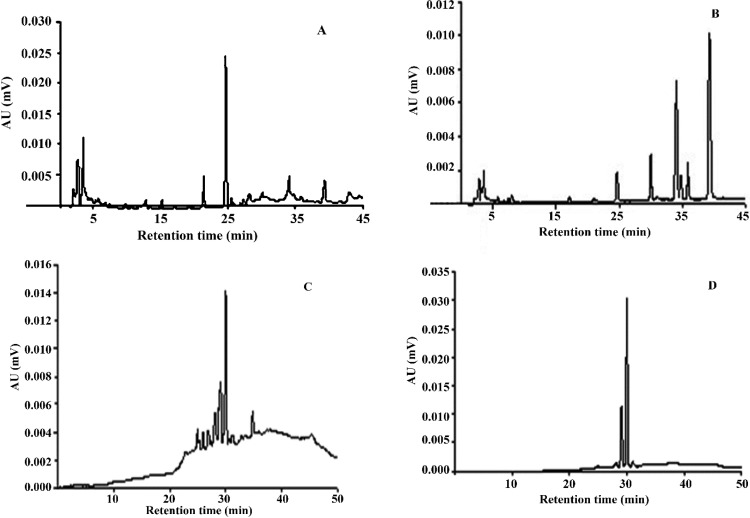
Chromatogram of *P. carthagenensis* (**A**), *P. capillacea* (**B**), *P. leiocarpa* (**C**) and *P. deflexa* (**D**) extracts by HPLC/PDA analysis.

After determination of the antioxidant potential and levels of constituents, we analyzed the methanolic extracts obtained from *Psychotria* in an analytical LC (Figure 3). The standards were easily identified based on their UV absorption spectra and retention times. The substances found in the extracts were unambiguously identified by performing co-injection experiments in which aliquots of extract and standard were mixed and diluted to a known volume, and analyzed by HPLC. Only *p*-coumaric acid (*tr* = 24.91 min) was found in *P. carthagenensis* and *P. capillacea* (Figure 3A,B).

The antioxidant activity of the pure *p*-coumaric acid at 40 μg/mL concentration was determined. DPPH radical scavenging activity of *p*-coumaric acid was found to be 55.6% ± 3.4%; the β-carotene bleaching with 78.6% ± 2%; and 87.14% ± 5.7% in ABTS radical scavenging activity. Comparison of the obtained of *P. carthagenensis* and *P. capillacea* data indicated the potent activity for the pure *p*-coumaric acid.

*p*-Coumaric acid (4-hydroxycinnamic acid), a phenolic acid, hydroxyl derivative of cinnamic acid, is widely used in the chemical, food, health, cosmetic, and pharmaceutical industries. Studies reported that antioxidants, such as *p*-coumaric acid and others hydroxycinnamic acids, function as chemoprotective agents by quenching carcinogenic nitrosating agents in several biological compartments, including salivary and gastric fluids [48]. However, minor attention has also been directed to the activity of simple phenolic acids, such as benzoic or cinnamic acids, and their derivatives.

## 4. Conclusions

According to data obtained from the present study, *P. carthagenensis* and *P. capillacea* were found to be effectives antioxidants in different *in vitro* assay including DPPH radical, ABTS radical and β-carotene bleaching activities when they are compared to standard antioxidant compounds, such as BHT, gallic acid, quercetin and ascorbic acid. The results indicate that the antioxidant activity to these plants can be attributed the presence the *p*-coumaric acid. Further, more detailed, studies on the chemical composition of those extracts, as well as studies with other models, such *in vivo* assays, are essential to characterize them as biological antioxidants.
